# Being there: A scoping review of grief support training in medical education

**DOI:** 10.1371/journal.pone.0224325

**Published:** 2019-11-27

**Authors:** Laura Sikstrom, Riley Saikaly, Genevieve Ferguson, Pamela J. Mosher, Sarah Bonato, Sophie Soklaridis

**Affiliations:** 1 Office of Education, Centre for Addiction and Mental Health, Toronto, Ontario, Canada; 2 Holland Bloorview Kids Rehabilitation Hospital, Toronto, Ontario, Canada; 3 Division of Child and Adolescent Psychiatry, Hospital for Sick Children, Toronto, Ontario, Canada; 4 Division of Psychosocial Oncology, Princess Margaret Cancer Centre, Toronto, Ontario, Canada; 5 Department of Psychiatry, University of Toronto, Toronto, Ontario, Canada; 6 Departments of Psychiatry and Family and Community Medicine, University of Toronto, Toronto, Ontario, Canada; 7 Cross-appointed Scientist, Wilson Centre, University Health Network and Faculty of Medicine, University of Toronto, Toronto, Ontario, Canada; Universitat d’Alacante, SPAIN

## Abstract

**Introduction:**

Medical education experts argue that grief support training for physicians would improve physician and patient and family wellness, and should therefore be mandatory. However, there is little evidence about the range of curricula interventions or the impact of grief training. The aim of this scoping review was to describe the current landscape of grief training worldwide in medical school, postgraduate residency and continuing professional development in the disciplines of pediatrics, family medicine and psychiatry.

**Methods:**

Using Arksey and O’Malley’s scoping review principles, MEDLINE, EMBASE, ERIC, PsychInfo and Web of Science were searched by a librarian. Two levels of screening took place: a title and abstract review for articles that fit a predefined criteria and a full-text review of articles that met those criteria. Three investigators reviewed the articles and extracted data for analysis. To supplement the search, we also scanned the reference lists of included studies for possible inclusion.

**Results:**

Thirty-seven articles published between 1979 and 2019 were analyzed. Most articles described short voluntary grief training workshops. At all training levels, the majority of these workshops focused on transmitting knowledge about the ethical and legal dimensions of death, dying and bereavement in medicine. The grief trainings described were characterized by the use of diverse pedagogical tools, including lectures, debriefing sessions, reflective writing exercises and simulation/role-play.

**Discussion:**

Grief training was associated with increased self-assessed knowledge and expertise; however, few of the studies analyzed the impact of grief training on physician and patient and family wellness. Our synthesis of the literature indicates key gaps exist, specifically regarding the limited emphasis on improving physicians’ communication skills around death and dying and the limited use of interactive and self-reflexive learning tools. Most trainings also had an overly narrow focus on bereavement grief, rather than a more broadly defined definition of loss.

## Introduction

Grief is a profound human experience. The loss of a loved one to death is the most recognized form of grief, but grief also emerges from the loss of home, family, function or ability [1]. Although most forms of grief resolve over time, intense feelings of loss and suffering can also trigger the onset of physical or mental health problems [1]. As well, the advent of medical technology capable of sustaining life without restoring health has also changed where, when and how we die [2]. For example, estimates suggest that between 30 to 90% of all deaths in North America and Europe occur in hospital [3]. The hospitalization of death and dying in the last five decades means that physicians are increasingly involved in the assessment and support of patients and caregivers experiencing a range of losses, but they often report feeling inadequately prepared to do so [4–10]. Given this information, it is striking that grief remains part of the “hidden curriculum” in medical education [11]. The lack of formal training opportunities for physicians reinforces the harmful idea that one must “soldier on and deal with it” rather than process or discuss distressing events [12,13]. The lack of attention paid to physicians’ personal or professional experiences with grief can result in burnout and depersonalized care [14–19] and the failure to train physicians appropriately may also result in misinterpreting or mistreating grief as stress, anxiety or depression [1]. Thus, the importance of providing physicians with the knowledge and skills to offer grief support to patients, bereaved caregivers and colleagues at critical moments cannot be understated.

The goal of our scoping review was to determine the range of training opportunities worldwide, identify where gaps in grief training exist and propose ways these gaps might be narrowed [20–23]. Our scoping review focused on grief and not depression. It is important to make a distinction between these two conditions since grief is a biophysical response to loss that often resolves itself, whereas depression is a psychiatric condition that requires treatment [24]. We identified all relevant literature on grief training related to teaching, learning and curriculum within the areas of pediatrics, family medicine and psychiatry. We chose these subspecialties because physicians who practise in these disciplines are often engaged with patients and their caregivers for prolonged periods. For example, most pediatricians keep in touch with grieving families: 71.9% have attended a patient’s funeral [25], 61% call the family and 68% initiate face-to-face follow-up consultations [26]. More importantly, bereaved family members find solace in these interactions [27]. Physicians from these subspecialties are also very likely to be members of interprofessional health teams that coordinate care in disciplines where death and dying are more sudden, including intensive care units or emergency departments.

In this article, we describe our scoping review methodology and describe the results of our data abstraction. Our data synthesis includes both quantitative and qualitative analysis. We describe the definitions of grief, rationale for providing grief support training, grief training formats, educational aims, teaching methods, grief training outcomes and challenges for implementing grief training. By surveying the state of medical education on grief support training, we identify strengths and gaps in the available curricula and literature and propose strategies for educational research and development.

## Methods

### Scoping review

Given the lack of curricular opportunities to learn about grief in medical school, residency and continuing professional development, we adapted Arksey and O’Malley’s scoping review methodology to capture the breadth of existing knowledge on grief and bereavement training in this field, as represented by all publications about research and curriculum design [20,21,23]. Below, we briefly present the five phases of this process. A more detailed review of our protocol is published elsewhere [22].

### Planning and research question

The planning phase of this study included a psychiatrist/paediatrician (PJM) and an education scientist (SS) who noted that medical trainees felt they did not know what to do or say after the death of a patient. These observations resulted in an environmental scan of the literature on grief support training in medicine, which indicated that few studies describe outcome or impact. Thus, a scoping review was favoured over a systematic review. Our research question for this review was: What is known from the existing academic literature about grief training in medical school, residency programs and continuing professional development in psychiatry, family medicine and paediatrics?

### Information sources

An experienced health science librarian (SB) conducted all of the literature searches in consultation with the research team. We created search strategies for five bibliographic electronic databases and for the concepts of grief, medical residents, postgraduate training and continuing professional development and education using a combination of keywords and controlled vocabulary terms applicable to each database. The keywords and controlled vocabulary terms searched were relevant to the concepts of medical residency (e.g., internship, residency), grief (e.g., grief, bereavement) and education (e.g., teaching, learning). A focused grey literature search of Biosis, Papersfirst and ProQuest (e.g., conference papers, dissertations) was combined with a scan of the reference lists of the included studies (S1 Table). All publication types (e.g., research studies, commentaries, evaluation reports, book chapters, curriculum descriptions) were eligible for inclusion. We did not limit the search by date. Our search was peer reviewed using the Peer Review of Electronic Search Strategies (PRESS) checklist [23,28]. We conducted the initial search on December 7, 2015 and a follow-up search on May 24, 2019.

### Screening

Two levels of screening took place: a title and abstract review for articles that fit the predefined criteria and a full-text review of articles that met those criteria. For first- and second-level screening, three authors (LS, SS and GF) screened all of the titles and abstracts independently. During second-level screening, the same authors reviewed the full-text articles to determine whether they met the inclusion criteria. A third reviewer (PJM) was consulted to resolve any discrepancies. For example, some end-of-life articles discussed the concept of grief but upon further consideration were excluded because there was no discussion about grief training for end-of-life discussions.

### Data abstraction

The review team summarized information from the selected articles and recorded relevant data in an Excel sheet, including the setting, medical discipline, stage of career, research design, results and recommendations of future research. We also followed Levac and colleagues’ recommendations for the charting process [20,21]. The review team tested a draft abstraction form on a random sample of 10 articles. Next, we collectively developed the data extraction form to include descriptive information) and thematic categories (e.g., definitions of grief, study methodology, educational aims) [29,30]. Differences in the data abstracted were resolved through discussion and columns were added or deleted from our spreadsheet as needed. As recommended by Arksey and O’Malley, we did not formally appraise the study methodologies since our aim was to identify and describe the existing literature on grief training in medical education.

### Synthesis

**Fig 1:**
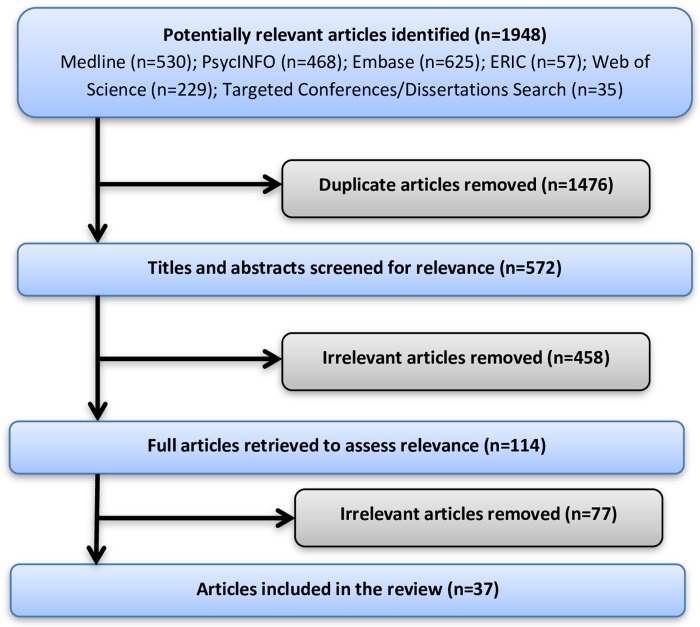
Scoping review process.

Data analysis involved both quantitative and qualitative synthesis by a multidisciplinary team. As stated, a librarian with methodological expertise in scoping reviews conducted the searches with input from the research team. A paediatrician/psychiatrist (PJM) ensured that the content and key terms were relevant. An anthropologist (LS) and an education scientist (SS) with expertise in qualitative data analysis completed the quantitative and thematic analysis. We used data abstraction sheets to tabulate and compare descriptive information and to gather frequency statistics (e.g., field of study in medicine, format of training). Then we used thematic analysis to generate open descriptive codes that emerged explicitly from the literature (e.g., purpose of grief training). Next, we synthesized these primary descriptive codes into axial codes. Axial coding in grounded theory is the process of relating categories and concepts to each other via inductive and deductive thinking [31,32]. Our axial codes reflected thematic similarities in both educational aims and learning methods from the reviewed literature (e.g., self-awareness). As a final step, we created a separate table to list gaps in research evidence, methodological approach and evaluation (Fig 1).

## Results

Our search produced 1948 potential articles for inclusion. After a three-part screening process, we found thirty-seven publications eligible for inclusion from 1979 to May 2019 (Fig 1). Of those we included, the majority (57%) described grief education in the United States (n = 21) and the United Kingdom (19%, n = 7). Two papers described grief education in Australia (n = 2). Single papers described and evaluated medical education in Canada, Germany, Greece, Denmark, Spain, Ireland and South Africa. One study compared and contrasted death education via anatomy classes at a Canadian/American medical school. Nearly half of the publications (49%, n = 18) were descriptions of curricula content, approach or learning methods. The other half (51%, n = 19) were research, including curricula evaluation studies (n = 9), exploratory studies (n = 6), randomized controlled trials (n = 3) and one investigative questionnaire-based study. The foci of these publications were undergraduate medical education (n = 17), followed by postgraduate or residency training (n = 11) and continuing professional development (n = 9). Although our search strategy was limited to paediatrics, psychiatry and family medicine, our results included seminars and workshops offered in emergency medicine, geriatrics and palliative care. We chose not to exclude these articles because family physicians, paediatricians and psychiatrists were often invited to these seminars and workshops as part of routine rotations during medical training or as a member of an interprofessional team. For a summary of our analysis see Table 1. For descriptive characteristics of the included literature, see S2 Table.

**Table 1:** Summary of the results.

Feature	No. (%)
Educational Setting	
Undergraduate	17 (46)
Residence	11 (30)
Continuing professional	9 (24)
Educational Aims	
Knowledge	29 (78)
Personal growth and self-awareness	15 (41)
Communication skills	15 (41)
Interprofessional collaboration	6 (16)
None stated	5 (14)
Teaching/Learning Methods	
Lectures/interactive seminars	21 (57)
Debriefing	16 (43)
Bereaved caregiver involvement	7 (19)
Audio-visual techniques	5 (14)
Writing exercises	5 (14)
Hospital and site visits	3 (8)
Disseminated reference materials	3 (8)
Simulation and role-play	3 (8)
Memorials	2 (5)
Case studies	1 (3)
None described	8 (22)
Evaluation Design	
None described and/or measured	16 (43)
Self-assessment questionnaire	17 (46)
Post	14 (38)
Pre/post	9 (24)
Randomized controlled trial	3 (8)
Longitudinal follow up*	4 (10)
Participatory evaluation	2 (5)
Qualitative assessment	2 (5)

*5, 6, 12 and 18 month follow ups were described

### Descriptive analysis

#### What is grief?

Our scoping review found that only 14% of articles (n = 6) provided a theoretical framework or empirical definition of grief [33]. Grief was described as a non-linear process that is experienced in “waves,” rather than a slow incremental improvement in symptoms [33–35]. Although rarely discussed explicitly, grief was also characterized as a process influenced by a number of social factors [35]. Cultural background, social class, religious beliefs, individual capacity to cope with stress and the role that the deceased held in the family were described as factors that influence grief trajectories. The focus of all of the articles was on grief related to bereavement, or the death of a loved one.

#### Rationale for providing grief support training

All the articles emphasized grief training as necessary at all stages of medical training, from undergraduate to postgraduate and in continuing professional development [33–36]. Most of the articles noted that grief from singular or cumulative losses may lead to mental health disruptions, including adjustment problems, anxiety (e.g., separation anxiety, procedural anxiety, medically-associated traumatization, social anxiety, generalized anxiety, panic attacks, obsessive-compulsive disorder), depression with occasional suicidality and conversion/somatic symptom disorders [37,38]. A few of the articles also reported that physicians experience overwhelming emotional reactions and stress responses to patient deaths, yet often feel a lack of collegial support that would help them cope [36,39,40].

Several authors found that health care professionals who care for children with life-threatening conditions have intense feelings of helplessness, guilt, anger, sadness and grief over the child’s circumstances [39–42]. One study of end-of-life care for children in Greece described how the death of a child is often considered a “triple failure” for physicians [43] because they lacked the means, skills or abilities to save a life; because as adults, they were unable to protect a child from harm and because they betrayed parents who trusted them with the most valuable person in their lives. The death of a patient can violate the physician’s concept of professional purpose, which may result in burnout after cumulative losses.

#### Grief training formats

Most of the articles described grief training within medical education as a single voluntary workshop or seminar series over the course of one’s formal education. Workshops and seminar series on grief were commonly characterized as either one- or two-day sessions, consecutive workshop series (maximum of one week) or short modules within existing curricula [5,33,34,36,44–54]. The majority of sessions were offered outside the core curriculum or as elective courses within undergraduate medical education. Many were advertised as interdisciplinary and included students from nursing, occupational therapy and social work [33,49,53,55]. At both the undergraduate and postgraduate levels, most of the training modules consisted of didactic teaching methods (e.g., lectures) paired with a variety of group activities, which are described in more detail below. Three studies also described the dissemination of information pamphlets, posters and reference materials on grief and depression [56–58]. These materials summarized the latest evidence and, in one case, were combined with voluntary study days or small group discussions and lecturers [58].

Across the literature, there was no consensus about when trainees should be exposed to grief support training. A few authors made compelling arguments to integrate grief training throughout the undergraduate and postgraduate medical curriculum [59–61]. For example, one article suggested that grief training should run concurrently with anatomy dissection labs to relieve potential anxiety, prevent depersonalization of care and ensure positive coping strategies are learned at the onset of training [59]. Another article presented findings from an integrated quality improvement program designed to increase physicians’ competence in caring for dying children. In this program, four interventions designed to foster interprofessional collaboration and support were integrated into routine clinical care (e.g., mandatory bereavement debriefing sessions after the death of a patient) [42]. In a third example, medical students shadowed a trauma chaplain for a day as an experiential learning opportunity [61].

#### Educational aims

Grief training emphasized a wide range of educational aims. The most common included increasing knowledge, building capacity for self-awareness and reflection, improving communication skills and working on interprofessional teams. We discuss each of these aims below.

#### Knowledge

All of the articles recognized the importance of transmitting specific grief-related knowledge to trainees. For example, several articles emphasized the importance of understanding the clinical, ethical, legal, professional, cultural, spiritual and emotional dimensions of grief and bereavement for participants [5,32,36,38,45,46,51]. The rationale for this approach was that increased knowledge about grief translated into better patient care. For example, by gaining knowledge about grief, physicians may identify cases of complicated grief more easily [55,60,62]. Alternatively, by gaining knowledge about multicultural burial practices, physicians may provide better care to bereaved family members from varying religious or ethnic backgrounds [36]. This was important as a tool to help physicians understand that some signs of grieving (e.g., anger or wailing) were not attacks on their profession or person [37]. Increased knowledge about issues related to death and dying were also considered crucial for understanding the legal and ethical dimensions of autopsy and organ transplantation and medically assisted death [33,47].

#### Self-awareness

The literature reviewed emphasized the importance of providing physicians with opportunities to understand and reflect on their own processes for dealing with grief to improve patient care and their own well-being (41%, n = 15) [33,38,59]. Greater self-awareness was considered a skill that would improve patient care since physicians would acquire insights into grief from the patient’s point of view [35,61,63]. Personal growth and self-awareness were considered necessary to help residents gain insight into the impact of patient deaths on their own emotions and to cope with stress and potential burnout [34,37,52,63].

#### Communication

Improving communication skills was a key aim of 41% (n = 15) of the articles reviewed. Specific aims included communicating medical information effectively, listening to the concerns of patients nearing end of life and the bereaved and responding to crises [49,57,60]. A workshop geared towards dealing with death and bereavement in emergency medicine included additional content focused on notifying family members of unexpected deaths (e.g., from suicide or accidental death) [45].

#### Interprofessional collaboration

Several articles described the importance of interprofessional collaboration. By the end of grief training, it was important that participants knew about teamwork, nursing care, drugs for relieving distress and different professional roles and how to access and coordinate with them [36,39,49,60,61]. For example, an interprofessional workshop in pediatrics deepened trainees’ understanding of the role of chaplains for families who are grieving and of multidisciplinary strategies to support a family after their child’s death [61]. Post-workshop evaluations of site visits to hospices or palliative care units also suggested that trainees felt these visits helped them better understand the medical care system, the extension of care beyond treatment of disease, symptom management and ways to communicate with families [42,44].

### Teaching/learning methods

In addition to the standard lecture and discussion format, a range of activities were included in the reviewed studies’ curricula. We discuss each of the following below: the inclusion of bereaved caregivers in content delivery, audiovisual materials and group discussion, self-reflection exercises, simulation and role-play, reference materials for self-directed learning and memorials.

#### Inclusion of family in content delivery

Family members were involved in grief training in various ways. In some articles, bereaved family members helped co-facilitate workshop sessions, panel discussions or small group discussions [36,48,50,51,63]. Other authors described family members’ participation in live interviews or small group discussions about their grief experiences, followed by larger group discussions [50,51]. The inclusion of bereaved caregivers served as a curricular tool to provide the perspective of, and improve communication skills with, bereaved caregivers and facilitate personal emotional growth of trainees. Trainees reported a high sense of value when patients or bereaved family members were engaged in the training [44,50,51,63].

#### Audiovisual material and small group discussions

Audiovisual materials were often used in lectures, seminars and/or workshops to impart new knowledge, illicit emotional responses or illustrate teaching points. Audiovisual material was described as an experiential learning device that required limited resources [63]. One article argued that film facilitated an understanding of the psychological dynamics involved in grief and demonstrated the range of human responses to death [33]. Videos were shown with trainees and faculty in attendance and were followed by discussion linked to clinical case material [35]. The actual content of the audiovisual materials varied. Goddell and colleagues described using a taped interview with a prominent physician in the final months of his life [46]. Feature length documentaries or films were used to increase participants’ comfort levels when talking about death [33,44] and video clips were used to model helpful communication techniques about how to approach neonatal death with families [33,50,61,64].

#### Self-reflection exercises

Self-reflection was described as a useful tool for learning about grief by compelling learners to examine the context, meaning and implications of their attitudes and experiences [36,48,63,65]. For example, Rosenbaum described an exercise where trainees were asked to visualize their own deaths and write essays reacting to course content [6]. In a similar workshop, residents explored their own experiences with grief in small group discussions to help them develop skills to support grieving patients and caregivers [35]. During “death rounds,” trainees described a palliative medicine situation with personal significance [44]. Each case was discussed by the entire group and an attempt was made to address the challenges faced by the student and the health care team. These articles also discussed ways that more senior physicians could share their own experiences with death and dying to role model communication, self-reflection and interprofessional collaboration in everyday clinical settings. Activities designed to reflect on and discuss grief with peers had a positive impact on trainees, encouraging them to delve more deeply into their experiences, feelings and concerns [39,59]. Opportunities to debrief in group sessions were also rated positively [59].

#### Simulation and role-play

Three studies described role-playing or simulation activities during workshops for grief training. The aim of these activities was to build competency around notifying families about a death, communicating difficult news and listening to the patient’s needs. For example, an article described trainees reenacting a play to illustrate how children’s roles change within a family following the death of a parent; videos of these activities enabled debriefing later [39]. In another example, instructors enacted two scenarios where they communicated bad news about the end of a patient’s life [45]. In one, the physician used common, yet ineffective, methods for delivering this news. In the second, he demonstrated more effective methods. These demonstrations were followed by a formal presentation of principles for effective delivery of bad news. Simulation training also enabled trainees to practise communication scenarios in child bereavement [48,50,54].

#### Reference materials

A small number of studies reported on the dissemination of reference materials that summarize best practices in end-of-life care, models of care for identifying complicated grief and online resources for further study or personal reflection. Reference materials included posters for doctors’ offices, pamphlets, an online education resource and preparation checklists [55,56,58].

#### Memorials

A small number of authors discussed rituals or memorials designed to commemorate the dead. For example, following anatomy class, trainees held a memorial service to celebrate the gift of the body with the extended family present [59]. Bereavement debriefing sessions after the death of a child also provided opportunities for physicians to make sense of the losses they experienced and to renew purpose in their work [42]. These sessions also included informal rituals of remembrance, such as sharing a poem or signing sympathy cards for bereaved family members.

### Grief training outcomes

The impact of grief training was measured in 57% of articles (n = 21). Most of these (81%) relied on self-assessments (e.g., Likert scale) that occurred immediately after the training was complete. We will discuss each of the main outcome measures: impact on patient care and impact on physician well-being.

#### Impact on patient care

Self-assessed knowledge outcomes indicated that most trainees reported increased comfort levels discussing loss and death with patients and families and increased knowledge about the importance of effective communication. Most participants also reported a deeper understanding of their personal beliefs and attitudes toward death and dying that might impact care [35,40,41,45,46,51,64]. However, very few studies measured the impact on patient care. A study measuring the impact of a national model of care for sudden infant death syndrome (SIDS) in Ireland found that, after the workshops, families were more likely to be given the opportunity to hold their infant, given more privacy and offered keepsakes; as a result, they reported higher satisfaction with the quality of care [58]. A randomized controlled trial (RCT) of grief support training in Denmark found that physicians identified more cases of complicated grief and prescribed fewer psychotropic drugs than those in the control group who did not receive any training [57].

Surprisingly, another RCT in Spain found the opposite; they reported that widows in the intervention group (i.e., their family physician was trained in bereavement care) experienced poorer emotional outcomes than the widows in the control group [62]. Bleeker and Pomerantz provided one explanation for this in their report on pre-/post-evaluations of a non-mandatory lecture series (thirteen two-hour sessions with a psychiatrist) on loss and grief for second year medical students (n = 117) [52]. They found that participant’s anxiety and negative attitudes about communicating about dying and their lack of knowledge about proper care of dying patients did not change after the lecture series, despite the positive acquisition of knowledge. They noted that, although it seemed counterintuitive, the more personal experience a trainee had with death, the more likely they were to avoid important conversations about death with patients and their caregivers. They recommended that all trainees be given time to reflect on their own feelings and experiences with death and dying.

#### Impact on physician well-being

Very few of the studies measured the impact on physician well-being. Debriefings that included the experiences of colleagues made people feel less isolated and more connected to their colleagues [40]. An RCT in the US noted significantly decreased levels of burnout (i.e., emotional exhaustion and depersonalization) after attending four modules on stress, grief and burnout in medicine [34]. These authors did not find an impact on physician well-being and hypothesized that grief requires more time to abate than stress or burnout.

### Challenges for implementing grief training

Multi-level challenges influenced the ability to incorporate grief training into medical education. Logistical challenges included the ability to create class sizes small enough to provide quality training on grief [47]. Two studies noted that without a faculty lead or champion, grief training activities were not sustained after staff turnovers [39,66]. At an institutional level, there were also varying degrees of support for grief-specific training within different universities as a result of competing curriculum needs [66]. It was noted that the motivation to introduce grief training may be low, as some physicians do not consider it part of their job description [45,52].

## Discussion

Our review identified 37 published sources on grief training for physicians starting in 1979; to our knowledge, this is the first review to investigate grief training in medical education since 1997 [67]. Despite the fact that many medical educators argued that grief support training should be mandatory, our synthesis found few identifiable opportunities for medical students, postgraduate residents or physicians to learn about grief. In addition, most workshops were voluntary, short and occurred in pre-clinical stages when participants had limited experience dealing with death and dying, which limited their ability to absorb new knowledge and then apply it in clinical practice [68,69]. The narrow focus on knowledge acquisition of the legal and logistical dimensions of end-of-life care also indicated that there were significant training gaps at all stages of learning.

It seems clear that personal experiences with death played an important role in attitude formation and that lectures alone were insufficient to transmit the importance of learning this information [70–81]. Opportunities to debrief and reflect with peers were highly valued by trainees but there were few opportunities for trainees to discuss and process their own experiences with loss [6,11,12]. Also, while most of the articles reviewed reported strong support for grief training at all levels within medical education, the actual impact on behaviour and practice in clinical settings was unclear since only a few (11%) of the studies measured longitudinal outcomes. For example, Gerhardt reported that although participants self-reported improved competence and comfort with palliative care, the workshop did not uniformly produce lasting improvements in knowledge [68]. They concluded that sustained knowledge would likely require more intensive training integrated throughout the medical curriculum.

Significantly, very few of the articles provided empirical definitions of the grief process [1]. O’Connor argued that, without continuing professional development, physicians may rely on outdated theories and constructions of grief, which could negatively impact patient care [5]. Moreover, few of the studies we found discussed grief training in continuing professional development; yet, ongoing training can foster and emphasize the need to improve communication skills and help mitigate burnout [34,41,42]. Moreover, only two of the studies measured the impact of grief training on patient care outcomes by talking to grieving families or patients [57,58,62]. More rigorous approaches to measuring the impact on patient care are also needed. As well, we found evidence that learning was amplified by integrating grief training into other areas of medical education, thereby providing opportunities to practise and successfully apply new skills [57]. Site visits, interprofessional collaboration and the direct involvement of dying patients and bereaved caregivers were highly valued by trainees across all levels of training [57,62,68]. Thus, grief support training in undergraduate medical education in particular may not adequately prepare trainees for the reality of working on interprofessional teams. Future studies should explore possibilities for interdisciplinary collaborations and experiential learning opportunities to facilitate the integration of grief training into medical training [69–73,75].

Finally, none of the studies explored ways that patients experience grief as they cope with a range of other losses (e.g., disability, disaster, advanced cancer, miscarriage) [82,83]. Given that scientific and medical advances over the last century have made it “easier” to live but “harder” to die—effectively making chronic care the norm in medicine—teaching physicians to help patients live until they die is a pressing concern [2,82,83]. Additionally, few of the articles described the importance of understanding the cultural aspects that might influence the process of grieving [84–87]. For example, through a cross-sectional survey of advanced cancer patients, researchers in Japan found that reassurance statements like “hope for the best and prepare for the worst” are highly valued by patients [87]. It is also well documented that hope is an effective therapeutic technique; by this, we mean that believing in a treatment’s efficacy has physiological and psychological effects (e.g., the placebo effect) [88–90]. Therefore, physicians and patients alike may deliberately avoid conversations about death and dying to improve patient outcomes [90]. Given the potential impact of frank discussions about death and dying with patients, these issues warrant further study. How physicians address their own needs for emotional support in the care of patients with life-threatening and life-altering conditions also merits further research [5–7].

### Study limitations

Although we sought to be as thorough as possible, the study is limited to the subspecialties’ of family medicine, psychiatry and pediatrics. Given the increasing realities of chronic care as medical advances extendlife, future studies comparing our results to other subspecialties, in particular internal medicine and geriatrics, would be valuable. Despite these limitations we believe our inclusion/exclusion criteria were effective for generating questions that are relevant to many subspecialties. In particular, the overly narrow focus of grief training on loss related to death and dying, rather than other losses that impact the quality of life and physician well-being are worth noting.

## Conclusions

Grief is ubiquitous in the practice of medicine, yet there is a curious lack of training for physicians about what grief is and how it might impact patient care and their own well-being [85–87]. Currently, there is a very narrow literature on grief training for physicians. The findings within the limited literature indicated that grief training was most often a voluntary component of the medical curriculum, despite the fact that many patients rely on physicians to help them with their grief [72,91,92]. Nevertheless, this review found evidence that trainees valued being given ample opportunity to reflect on their own attitudes towards death and dying in small group settings [31,70,71]. As well, a physician’s awareness of their own and their patients’ grief reactions may allow both to have a higher quality of care [73,89,90]. Thus, medical training should include grief training to help physicians prepare for loss and develop competency in dealing the range of losses that will inevitably occur.

## Supporting information

S1 Table Search strategy. (DOCX)

S2 Table Descriptive characteristics of the literature. (DOCX)
