# Supplementary material for: Being there: A scoping review of grief support training in medical education
Source: PLoS One. 2019 Nov 27;14(11):e0224325. doi: 10.1371/journal.pone.0224325 (PMC6880967; doi:10.1371/journal.pone.0224325)
Supplement: S2 Table — (DOCX) [file pone.0224325.s002.docx]

**S2 Table: Descriptive characteristics of the literature**

| Author(s), year of publication | Type of Article | Country | Format of training | Healthcare area | Discipline of authors |
| --- | --- | --- | --- | --- | --- |
| **Undergraduate** | | | | | |
| Alshehabi, M., et al. (2011) | Research-evaluation | USA | End Of Life discussions between first year medical students and family members of deceased hospice patients | General | Hospice & End of life care |
| Black D., et al. (1989) | Curriculum, descriptive | UK | One, 1.5 hour Seminar integrated into psychology course with role-play | General | Pediatrics |
| Bleeker, JAC., et al. (1979) | Research- pre-post evaluation | USA | Non-mandatory lecture series with film and video tapes | General | Clinical Psychiatry |
| Clark S. (1996) | Curriculum, descriptive | Australia | Multi-professional three-day course with site visit and student presentations | General | Community and Family Medicine |
| Engel GL. (1980) | Curriculum, descriptive | USA | Seminar with film and discussion | General | Psychiatry |
| Field, D., et al. (2002) | Research - explorative evaluation | UK | N/a  Provides summary of availability of palliative care in medical schools in UK | General | Palliative care |
| Furst BA. (2007) | Curriculum, descriptive | USA | Seminar with film and discussion | General | Psychiatry |
| Gibbins, J., et al. (2009) | Research-qualitative explorative | UK | N/a  Research | General | Pediatrics and oncology |
| Goddell BW., et al. (1981) | Curriculum-descriptive | USA | Three seminars with video, live interviews, and small group discussion | General | Internal Medicine |
| Kitzes, J., et al. (2009) | Research-pre-post evaluation | USA | Death Rounds sessions facilitated by attending clerkship director, chief residents, and a palliative care physician | General | Geriatric medicine and Preventive medicine |
| Marks, SC., et al. (1997) | Curriculum, descriptive | USA and Canada | Integrated into Anatomy and Dissection lab | General | Cell Biology, Radiology, and Surgery |
| Mason C., et al. (1986) | Curriculum- evaluation | UK | Six seminars with  role-play, interviews, and video demonstrations | General | Community Medicine and Psychiatry |
| Perechocky A., et al. (2014) | Curriculum-descriptive | USA | Medical students shadowed a trauma chaplain | General | Emergency and General Medicine |
| Rosenbaum ME., et al. (2005) | Research-explorative evaluation | USA | Lecture-based course with film and four distinct self-reflection exercises | General | Medicine and Pediatrics and  Community and Behavioural Health |
| Smith, AM. (1994) | Questionnaire | UK | N/a  Provides summary of availability of palliative care in medical schools in UK | General | Palliative Medicine |
| Wittenberg-Lyles, EM. (2011) | Curriculum – descriptive | USA | Seminar Lecture style with inclusion of bereaved caregivers to present in small group format | General | Communication studies and  Palliative care medicine |
| Woolsey, SF. (1985) | Curriculum – descriptive | USA | Two-day elective course with lecture, group discussion and live interviewing with HCPs | General | Pediatrics |
| **Post-graduate** | | | | | |
| Carreno, M., et al. (2014) | Curriculum, descriptive | USA | Case studies | General | Geriatrics |
| Bagatell, R., et al. (2002) | Research, pre-post evaluation | USA | 6 discussion based seminar series | Pediatrics | Pediatrics |
| Dyer KA., and Thompson CD. (2000) | Research, statistical/evaluation | USA | An online medical education resource. [www.journeyofhearts.com](http://www.journeyofhearts.com) | General | Family medicine, psychiatry |
| Garcia JA., et al. (2013) | Research, cluster randomized control trial | Spain | 56 hours of primary bereavement care training over 30 months | General | Family Physicians |
| Gerhardt CA., (2009). | Research-pre-post | USA | One-day lecture-based workshop | Pediatrics and hematology/oncology | Biobehavioural health, psychology, pediatrics, pediatric psychiatry |
| Geschiere A., et al. (2018) | Research-qualitative | USA | Two 2-hour educational sessions | Family Medicine, Internal Medicine | \| Social work, Internal Medicine, Psychology \| \| --- \| |
| Jellinek MS., (1993) | Research-qualitative explorative | USA | Not described | Pediatrics | Pediatric intensive care |
| Ogle KS., et al. (2005) | Research - evaluation | USA | Survey to determine what training existed for EOL in post graduate education in USA | General | Family and community medicine and Medical Education |
| Menden M., et al. (2011) | Curriculum-descriptive | UK | Five-day training course built around experiences of bereaved parents | Pediatrics | Pediatrics |
| Schiffman JD., et al. (2008) | Curriculum-descriptive | USA | Six-week 1hr long course for residents | Pediatrics | Pediatric medicine , Child Psychiatry, and Pediatric Hematology-Oncology |
| Serwint,JR., et al. (2002) | Curriculum-descriptive | USA | One –day seminar with use of video, simulation and inclusion of bereaved family members | Pediatrics | Pediatrics |
| **Continuing Professional Development** | | | | | |
| Brysiewicz, P., et al. (2006) | Research, explorative | South Africa | Four year action research study to develop a model to deal with sudden death | General | Nursing, Social Work |
| Guldin MB., et al. (2013) | Research, cluster-randomized controlled trial | Denmark | Information pamphlets to both GPs and patients | General | Family Medicine |
| McDonnell, M., et al. (1999) | Research – pre-post evaluation | Ireland | Reference manual, study days, posters, phone hotline | General | Pediatrics, Family Medicine |
| Mediasauskaite A., and Kamau C., (2019) | Research , randomized controlled trial | UK | Four modules on grief, burnout, stress and loss | General | Occupational Psychology |
| Muthny, FA., et al. (2006) | Research, Post Evaluation | Germany | 1 day voluntary workshop | General, ICU physicians and nurses | General Practitioners, Internal Medicine |
| Papadatou, D. (1997) | Curriculum-descriptive | Greece | 600 hours of training over eight months | Pediatrics, nursing, social work, psychology | Nursing |
| Hylton Rushton, C., et al. (2006) | Curriculum-descriptive | USA | Four quality improvement interprofessional interventions integrated into general practice | Family medicine, nursing, social work, pediatrics, psychiatry | Pediatrics |
| Smith TL., et al. (1999). | Curriculum – descriptive | USA | Eight modules lasting 45-50 minutes each; delivered in a two-day workshop | General, Emergency Medicine | Emergency Medicine |
| Wayne Wolfram R., et al. (1998) | Curriculum descriptive | USA | A 50 minute “coping with the death of a child” module | Pediatrics | Pediatrics |
